# Obesity impairs male fertility through long-term effects on spermatogenesis

**DOI:** 10.1186/s12894-018-0360-5

**Published:** 2018-05-16

**Authors:** Yan-Fei Jia, Qian Feng, Zheng-Yan Ge, Ying Guo, Fang Zhou, Kai-Shu Zhang, Xiao-Wei Wang, Wen-Hong Lu, Xiao-Wei Liang, Yi-Qun Gu

**Affiliations:** 1 Lanzhou University Second Hospital, Lanzhou, 730020 People’s Republic of China; 2 Xiyuan Hospital, China Academy of Traditional Chinese Medicine, Beijing, 100091 People’s Republic of China; 3 Graduate School of Peking Union Medical College, No. 9 Dongdansantiao, Dongcheng District, Beijing, 100730 People’s Republic of China; 4 National Health and Family Planning Key Laboratory of Male Reproductive Health, Department of Male Clinical Research, National Research Institute for Family Planning, Beijing, 100081 People’s Republic of China

**Keywords:** Obesity, Spermatogenesis, Sex hormone-binding globulin, Testosterone

## Abstract

**Objective:**

This study aimed to investigate the effect and possible underlying mechanisms of high-fat diet-induced obesity on spermatogenesis in male rats.

**Methods:**

A total of 45 male rats were randomly divided into control (*n* = 15, normal diet) and obesity groups (*n* = 30, high-fat diet) and were fed for 16 weeks. Body weight and organ indexes were determined after sacrifice. Indicators of reproductive function, including sperm count, sperm motility, apoptosis of spermatogenic cells, and oxidative stress levels, were measured. Serum metabolic parameters and reproductive hormones were also assayed.

**Results:**

Compared with the control group, epididymal sperm motility in the obese rats was significantly decreased (*P* < 0.01). Morphological analysis of the obesity group showed vacuolar changes in seminiferous tubules, spermatogenic cell dysfunction, and increased apoptosis of spermatogenic cells in testicular tissue (*P* < 0.05). The calculated free testosterone (cFT) concentration in serum was decreased (*P* < 0.05), whereas the serum sex hormone-binding globulin (SHBG) level was significantly increased (*P* < 0.01). The superoxide dismutase (SOD) concentration decreased and the malondialdehyde (MDA) concentration increased in testis tissues; however, neither changes were statistically significant (*P* > 0.05).

**Results:**

Nutritional obesity can damage spermatogenesis in male rats due to long-term effects on spermatogenesis.

## Background

Obesity refers to excessive accumulation of body fat, which has a negative impact on health. According to the World Health Organization (WHO), a body mass index (BMI) of 25–29.9 kg/m^2^ is defined as overweight, while a BMI of 30 kg/m^2^ is defined as obesity. The incidence of overweight and obesity has sharply increased [1]. Relevant statistical data show that the obese population has doubled worldwide from 1980 to 2008, and more than 10% of the population is obese [2]. The prevalence of obesity has continued to rise according to subsequent surveys, with the most recent estimate indicating that 35.2% of men and 40.4% of women are obese [3].

Obesity is well documented to be linked to diseases such as type 2 diabetes mellitus, cardiovascular disease, cancers, and sleep apnea syndrome [4, 5]. Recently, the effect of obesity on fertility has been extensively investigated. However, current studies have principally focused on the effects of obesity on the reproductive function of females or female animals, while males or male animals are poorly studied [6]. Additionally, while most reports have examined obesity in relation to reproductive function, the underlying mechanisms have not been elucidated [7, 8]. In addition, conclusions on the effects of obesity on semen parameters and reproductive hormones have differed [9] due to the many factors that may impact male fertility. For example, a meta-analysis performed by MacDonald et al. found no statistically significant association between BMI and semen parameters [10], while a study performed by Sermondade et al. found a significant J-shaped association between BMI and an abnormal sperm count [11]. This study aimed to investigate spermatogenesis in male rats with obesity induced by high-fat diet administration to minimize experimental bias and identify possible mechanisms.

## Methods

### Animals

Six-week-old male Sprague-Dawley rats were provided by Vital River Laboratory Animal Technology Co., Ltd. (Beijing, China). The rats had a body weight of 130.43 ± 7.15 g. They were maintained on a 12-h day/12-h night schedule (lights on from 19:00 to 07:00 h). Temperature and humidity were maintained at 22 ± 1 °C and 60%, respectively. Food and water were provided ad libitum, and each cage contained 5 rats. We tried our best to minimize animal suffering, and CO_2_ inhalation was used for euthanasia.

### Experimental design

A total of 45 male rats were enrolled in this study and randomly divided into two groups, namely, the control group (*n* = 15, normal diet) and the obesity group (*n* = 30, high-fat diet), which received a normal diet and a high-fat diet, respectively. The high-fat formula was as follows [12]: 10% lard oil, 10% sucrose, 1.5% cholesterol, 0.5% bile salt, 5% yolk powder, and 73% normal feed. The body weights and lengths of the rats were measured each week for 16 successive weeks. At the 16th week, the rats in the lower quartiles for weight gain (*n* = 8) were excluded from the obesity group. As the rats with body weights in the bottom quartile tended to exhibit obesity resistance, they were also excluded, and 22 obese rats were ultimately analyzed. To ensure appropriate grouping, a comparison of general status was performed between the two groups. The general growth status and metabolic parameters of the two groups were compared at the end of the 16th week.

After being fed with the respective diets for 16 weeks, all rats were anesthetized with CO_2_. Blood samples were obtained from the abdominal aorta, centrifuged (2400 rpm for 20 min at 4 °C and frozen at − 70 °C) and were used to measure serum hormone levels [luteinizing hormone (LH), total testosterone TT), estradiol (E_2_), and sex hormone-binding globulin (SHBG)]. Testicular histology, apoptosis of spermatogenic cells, and antioxidant status of the testis tissues were analyzed. Sperm suspensions from the cauda epididymis were used to determine sperm counts and motility.

### Body index and organ index assessments

Bilateral testes, bilateral epididymides, and visceral fat (surrounding the kidney, testicles and omentum majus) were obtained and weighed. The relevant parameters were determined as follows: Lee index = [weight (g) × 10^3^/body length (cm)]^1/3^; fat coefficient = [visceral fat weight (g) × 100%/body weight (g)]; and testicular coefficient = [testicular weight (g) × 100%/weight (g)].

### Serum biochemical and hormone assays

Serum SHBG was determined using an enzyme-linked immunosorbent assay (ELISA) kit obtained from Beijing Northern Biological Technology Research Institute (Beijing, China). Serum LH, T, and E_2_ were measured with radioimmunoassays (RIAs) using a kit obtained from Beijing Northern Biological Technology Research Institute (Beijing, China). Serum free testosterone (FT) was calculated using the Vermeulen formula:

$$ cFT=\left[T-23.43 FT\right]/\left[ SHBG-\left(T-23.43 FT\right)\right]\times {10}^{-9} mol/L. $$

### Histological examination

Small pieces of testis were fixed in Bouin’s solution and 70% ethanol, dehydrated in graded ethanol, fixed with 10% formalin solution for 48 h, and treated with mixed decalcifying fluid. After dehydration with alcohol, the femoral heads were embedded with paraffin, cut into 5-μm sections, and stained with HE. Cell morphology was observed under a light microscope and evaluated with Johnsen scoring [13, 14]. The histological criteria for modified Johnsen scoring are as follows: full spermatogenesis (score 10), slightly impaired spermatogenesis, many late spermatids, disorganized epithelium (score 9), less than five spermatozoa per tubule, few late spermatids (score 8), no spermatozoa, no late spermatids, many early spermatids (score 7), no spermatozoa, no late spermatids, few early spermatids (score 6), no spermatozoa or spermatids, many spermatocytes (score 5), no spermatozoa or spermatids, few spermatocytes (score 4), spermatogonia only (score 3), no germinal cells, Sertoli cells only (score 2), and no seminiferous epithelium (score 1).

### Sperm count and motility

Sperm from the right cauda epididymis with a length of 1.5 cm were obtained. Internal rinsing with 1.0 ml of modified M199 medium was performed at 37 °C, and the samples were incubated for 30 min in a 37 °C water bath with vibration to induce the sperm to swim. Then, a 15-μl sperm suspension was extracted for sperm count and motility analysis using a Hamilton-Thorne Sperm Analyzer (HTM-IVOS). The sperm count per ml in the suspension from a unilateral epididymis was then calculated.

### Assessment of spermatogenic cell apoptosis

The TUNEL method was used to label the 3'-end of fragmented DNA in the apoptotic spermatogenic cells. The procedure was performed using Roche’s TUNEL chemical staining method (Roche, SWISS, Cat. No. 11684817910) [15]. The brownish-orange particles in the cell nucleus observed under a microscope were classified as apoptotic cells. The apoptosis index (AI) was determined as follows: 500 cells were selected from five high-power fields from each section, and AI = apoptotic cell number/500 × 100%.

### Antioxidant status evaluation

The kits for assessing testis antioxidation status, including superoxide dismutase (SOD) [16] and malondialdehyde (MDA) [17] concentrations, were purchased from Nanjing Jiancheng Bioengineering Institute (Nanjing, China). Testis tissues were isolated and crushed with liquid nitrogen, a homogenate was prepared, and the remaining procedures were performed following the kit protocols.

### Statistical analysis

Statistical analysis was performed using the SPSS 13.0 software package (Chicago, IL, USA). Student’s t-tests or Mann-Whitney tests were used to compare the results between the two groups. The results are presented as the mean ± s.e.m. in all cases, and *P* < 0.05 was considered statistically significant.

## Results

### General growth status and metabolic parameters

The results showed that compared with those in the control group, the Lee index and fat coefficient level of the nutritional obesity rats were significantly increased (*P* < 0.05), whereas the testis coefficient was significantly decreased (*P* < 0.05). No obvious difference was found in body length between the two groups, indicating that the group division was reasonable (Table 1).

**Table 1:** General growth status and metabolic parameters

	Weight	Length	Lee index	Fat coefficient	Testis coefficient
	(g)	(cm)	(%)	(%)	(%)
Control	552.90	26.50	0.31	4.38	0.63
*n* = 15)	±7.76	±0.94	±0.01	±0.96	±0.07
Obesity	619.80	26.86	0.32	5.02	0.55
(*n* = 22)	±7.56	±0.58	±0.01	±0.71	±0.06
*P* value	0.000^**^	0.198	0.046^*^	0.039^*^	0.001^**^

The data are presented as the mean ± s.e.m. ^*^ *P* < 0.05, ^**^P < 0.01: statistical significance compared with the control group; Weight: body weight; length: body length; Lee index = [weight (g) × 10^3^/Body length (cm)]^1/3^; Fat coefficient = [visceral fat weight (g) × 100%/body weight (g)]; Testicular coefficient = [testicular weight (g) × 100%/weight (g)]; and s.e.m.,: standard error of the mean

### Sperm concentration and motility

**Fig. 1:**
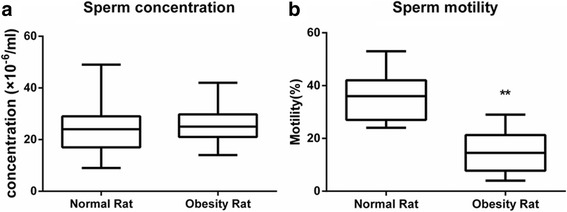
Comparison of sperm concentration (**a**) and motility (**b**) between the two groups. The data are presented as the mean ± s.e.m.; * *P* < 0.05 and **P < 0.01

To clarify the effect of obesity on the number and viability of sperm, sperm concentration and motility were detected. The concentrations of sperm extracted from the epididymis were 23.40 ± 9.72 × 10^6^/ml and 24.64 ± 7.16 × 10^6^/ml in the two groups, with no significant difference (*P* > 0.05). As illustrated in Fig. 1, sperm motility [(36.40 ± 9.17)% vs (14.36 ± 7.67)%] was significantly decreased in the obesity group compared with the control group (*P* < 0.01). The results indicated that obesity did not reduce the number of sperm but caused a marked decline in sperm viability.

### Histological examination results

**Fig. 2:**
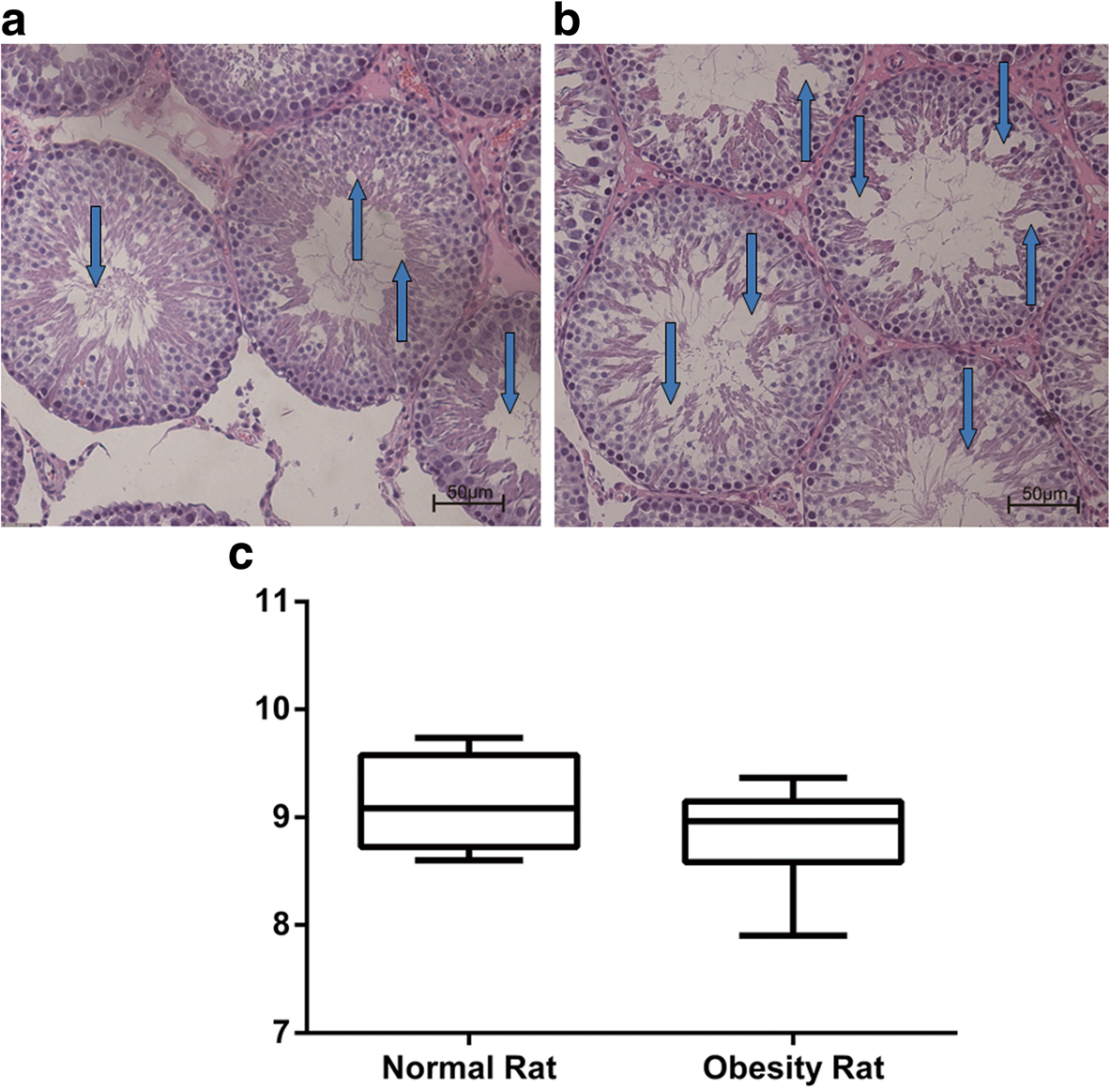
HE staining of testicular tissues (× 20) and comparison of sperm Johnsen scores between the two groups. **a** control group; **b**, obesity group; **c** the data are presented as the mean ± s.e.m.; * *P* < 0.05 and **P < 0.01

To assess the morphology of spermatogenesis and spermatogenic cells, histological examination was performed. HE staining demonstrated vacuolation in the seminiferous tubules and structural dysfunction in the spermatogenic cells or detachment of germ cells from the basement membrane in the obesity group (Fig. 2a, b). No significant difference was found in Johnsen scores [(9.14 ± 0.14) vs (8.86 ± 0.09)] (*P* > 0.05) between the two groups (Fig. 2c). We concluded that the short-term influence of obesity on testicular function may not be very obvious; however, long-term effects may damage spermatogenic function.

### Ai

**Fig. 3:**
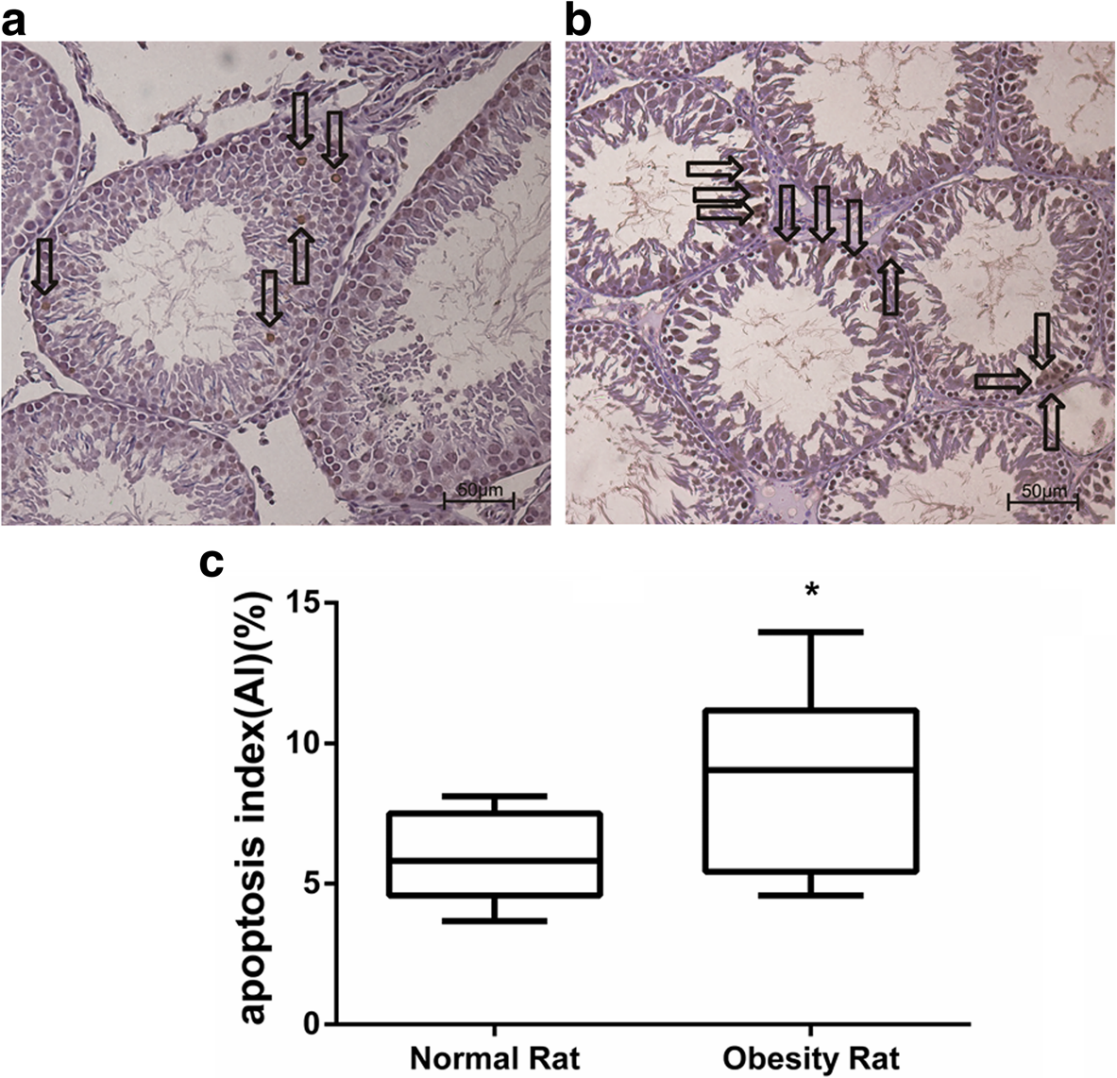
Apoptosis of spermatogenic cells in testicular tissues and comparison of the apoptosis index between the two groups (× 20, arrows indicate apoptotic cells). **a** control group; **b** obesity group; **c** the data are presented as the mean ± s.e.m.; * *P* < 0.05 and **P < 0.01

To observe the effects of obesity on spermatogenic cell apoptosis, TUNEL assay was performed. The AI of spermatogenic cells (Fig. 3a, b) was significantly increased in the obesity group compared with the control group [(5.95 ± 0.49)% vs (8.61 ± 1.05)%] (*P* < 0.05) (Fig. 3c). The results suggest that obesity may promote testicular germ cell apoptosis.

### Reproductive hormone assays

Regarding the effect of obesity on male reproductive hormones, serum cFT concentrations were decreased, and serum SHBG levels were increased (*P* < 0.05) in the obesity group. Although serum T levels decreased in the obesity group, no statistically significant differences were found between the two groups (Table 2).

**Table 2:** Reproductive hormone levels in the two groups

	LH	TT	E_2_	SHBG	cFT
	(mIU/ml)	(ng/ml)	(pg/ml)	(nmol/L)	(ng/ml)
Control	3.44	0.80	4.49	50.35	0.011
(*n* = 15)	±2.75	±0.76	±3.60	±6.26	±0.010
Obesity	3.20	0.59	4.39	85.13	0.003
(*n* = 22)	±2.76	±0.51	±2.45	±9.13	±0.002
P value	0.799	0.356	0.926	0.000^**^	0.031^*^

The data are presented as the mean ± s.e.m. ^*^ *P* < 0.05, ^**^P < 0.01: statistical significance compared with the control group; *LH* luteinizing hormone, *TT* total testosterone, *E2* estradiol, *SHBG* sex hormone-binding globulin, *cFT* calculated free testosterone, and *s.e.m*. standard error of the mean

### Oxidative stress assessment

**Fig. 4:**
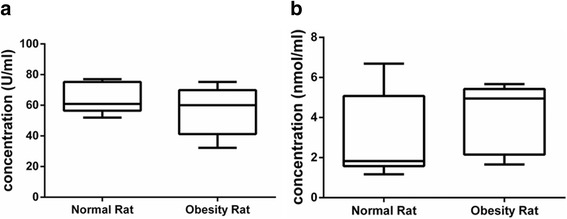
Comparison of the concentrations of SOD (**a**) and MDA (**b**) in the testis homogenate between the two groups. SOD: superoxide dismutase, MDA: malondialdehyde; the data are presented as the mean ± s.e.m.; * *P* < 0.05 and **P < 0.01

To determine the effect of obesity on oxidative stress, the concentrations of SOD and MDA in the testis homogenate were determined. The SOD and MDA concentrations of the testicular homogenate were [(64.8 ± 10.2) vs (56.6 ± 14.4) U/ml] and [(3.0 ± 0.7) vs (4.0 ± 0.8) nmol/ml] in the two groups (*P* > 0.05) and (*P* > 0.05), respectively (Fig. 4). The results indicated that the SOD concentration decreased while the MDA concentration increased; however, neither change was statistically significant.

## Discussion

Obesity is a chronic metabolic disease caused by interactions of various genetic and environmental factors. Interestingly, in the past 50 years, along with the annually increasing trend in human obesity, fertility has shown a parallel decreasing trend [18]. Several studies have indicated that a higher BMI is associated with significant decreases in sperm concentration [19–21]. Investigators have attempted to investigate the relationship between obesity and fertility decline; however, no consensus has been reached.

Currently, no unified standard is available for evaluating obese rats. In 1929, MO Lee [22] proposed the Lee index to evaluate obesity in rats, which is the most commonly used method to evaluate obese rats. In a diet-induced model of obesity reported by Levin, rats became obesity-resistant with high-fat diet administration [23], and differences were found in their energy, endocrine, fat and glutamic acid (GLU) metabolism. Based on the relevant literature and the Lee index, one-fourth of the rats in the high-fat diet group were excluded in our study because they were obesity-resistant. The remaining three-quarters of the rats were classified as the obesity group [24]. Our results demonstrated successful generation of the obese rat model.

Currently, the effect of male obesity on sperm count, motility, and morphology in humans is controversial. A review found that up to 2015, progressively reduced motility was reported in 13 of 35 articles, while a decreased sperm count with normal morphology was reported in only 9 of 29 papers [6]. However, two recent meta-analyses including 14 and 21 studies demonstrated an increased risk of azoospermia or oligozoospermia in overweight or obese males [25, 26]. The results from this study indicated that obesity induced by a high-fat diet can change the histomorphology of seminiferous tubules, which may not have obvious effects on male fertility immediately, but the long-term effects on spermatogenesis induced by obesity may impair male fertility.

Due to gene polymorphisms in the population, many factors (e.g., smoking, alcohol consumption, and medication use) that can affect seminal parameters are often overlooked. In addition, sample selection bias can lead to inconsistent conclusions regarding the impact of obesity on sperm. This study, by controlling various confounding factors, revealed that obesity reduced sperm motility without changing the sperm count, which is consistent with previous reports [25]. The possible mechanism may be damage to the integrity of sperm cell membranes, sperm cell DNA, and sperm mitochondria induced by excessive reactive oxygen species (ROS) [27, 28].

Apoptosis is an autonomous programmed cell death process that is stimulated under specific conditions and is regulated by various genes. We found that the AI of the spermatogenic cells increased significantly in the obesity group. A recent study found that apoptosis of testicular spermatogenic cells is one of the major causes of male subfertility [29]. Cell apoptosis is predominantly regulated and controlled by the homeostasis of Bax and Bcl-2. When the Bcl-2/Bax ratio is disrupted, downstream caspase signaling pathways are activated, resulting in apoptosis. A high-fat diet has been reported to increase Bax and caspase-3 expression but reduce Bcl-2 expression in the testis [30]. Therefore, based on our results, obese rats exhibited increased spermatogenic cell apoptosis due to imbalances of Bcl-2/Bax. Furthermore, obesity resulted in lipid metabolic disorders and hyperlipidemia, which may increase the stress response of the endoplasmic reticulum. Therefore, the incidence of spermatogenic cell apoptosis is further increased [31, 32] via increased GRP78 mRNA and protein expression.

Mammalian reproductive function is predominantly controlled and regulated by the hypothalamus-pituitary-testis (HPT) axis. Male reproductive endocrinology is principally composed of three groups of hormones, including GnRH, GnIH, LH, FSH, and T. T is one of the major sex hormones in males and has an important role in the HPT axis. Hypothalamic hormones are intricately associated with obesity-induced physiological changes, indicating a mutual cause-effect relationship between obesity and gonadal hormone decline [33]. In a study with 3219 European males, Fui and other investigators found that the TT and FT levels in obese males were lower than those in normal-weight males. Another study with 314 Asian males reached the same conclusion [34]. Camacho found that obesity reduces testosterone, low testosterone levels can promote male obesity, and testosterone increases after weight loss [35]. Compared with rats fed a normal diet, rats fed a high-fat diet had lower testosterone levels. When these rats were fed a normal diet, their testosterone levels returned to normal [35]. Although biologically active serum FT only accounts for 2% of TT, it is directly involved in functional activities, such as the development of male secondary sex characteristics, maintenance of spermatogenesis and sexual desire [36]. As a diagnostic marker for male hypogonadism, the serum cFT level exhibits better sensitivity than the serum TT level [37].

SHBG is a blood transport protein for testosterone and estradiol. Its synthesis and secretion are regulated by androgen and estrogen. Serum SHBG may exert direct or indirect effects on androgen conversion and metabolism, and it regulates GLU homeostasis and fatty acid metabolism. In this experiment, our results confirmed decreases in serum TT and cFT levels and an increase in the serum SHBG concentration in the obese rats. Therefore, we hypothesize that serum SHBG is the key factor in reducing serum cFT levels, and obese rats may develop mild primary hypogonadism (reduced serum TT and cFT, increased SHBG). Primary hypogonadism in the obese rats is likely the initiating factor for alterations in the HPT axis.

Oxidative stress is highly correlated with a wide variety of inflammatory and metabolic disease states, including obesity. Oxidative stress is highly correlated with cumulative damage in the body induced by free radicals that are inadequately neutralized by antioxidants, and oxidative damage is aggravated by decreases in antioxidant enzyme activities, such as those of SOD, catalase (CAT), and glutathione S-transferase (GST) [38]. Evidence suggests that there are many sources of oxidative stress in obesity [39]. In our study, to determine the effect of obesity on oxidative stress, the concentrations of SOD and MDA in testis homogenate were determined.

SOD, which protects cells from free radical damage by ROS, is an important enzyme that protects against injuries caused by internal and external superoxide ions. MDA is an aldehyde generated in the process of lipid peroxidation caused by free radicals. MDA indicates cell membrane damage and reflects the severity of an oxygen radical attack on reactive cells and the levels of free radical metabolism in vivo. Decreased SOD and increased MDA can trigger oxidative stress, causing cell damage and even death. This study found decreased SOD levels and increased MDA levels in the testicular tissues of obese rats, demonstrating that the oxidative stress level of testis tissues in obese rats is increased and may impact sperm motility. Several possible explanations may account for these findings: obesity is associated with elevated serum free fatty acids, and unsaturated fatty acids are susceptible to attacks by ROS, producing peroxidation and subsequently resulting in decreased SOD levels and MDA accumulation, which is ultimately reflected by an increased oxidative stress level [40].

## Conclusion

The rats with obesity induced by high-fat diet administration exhibited lipid metabolism dysfunction and altered reproductive hormone levels as well as increased oxidative stress levels in testis tissues, leading to mild primary hypogonadism. Meanwhile, the normal function of the HPT axis is maintained in the short-term through a corresponding feedback mechanism. However, the long-term effects of obesity may cause a decline in male fertility.

## Abbreviations

AI: apoptosis index
BMI: body mass index
cFT: Calculated free testosterone
CVD: Cardiovascular disease
DM: Diabetes mellitus
E_2_: Estradiol
FT: Free testosterone
HPT: Hypothalamus-pituitary-testis
HTM-IVOS: Hamilton-Thorne Sperm Analyzer
LH: Luteinizing hormone
MDA: Malondialdehyde
ROS: Reactive oxygen species
SHBG: Serum sex hormone-binding globulin
SOD: Ssuperoxide dismutase
SPF: Specific-pathogen-free
T: Testosterone
TT: Total testosterone
WHO: World Health Organization
